# ^89^Zr-trastuzumab PET supports clinical decision making in breast cancer patients, when HER2 status cannot be determined by standard work up

**DOI:** 10.1007/s00259-018-4099-8

**Published:** 2018-07-30

**Authors:** Frederike Bensch, A. H. Brouwers, M. N. Lub-de Hooge, J. R. de Jong, B. van der Vegt, S. Sleijfer, E. G. E. de Vries, C. P. Schröder

**Affiliations:** 1 Department of Medical Oncology, University of Groningen, University Medical Centre Groningen, Hanzeplein 1, Groningen, 9713 GZ The Netherlands; 2 Department of Nuclear Medicine and Molecular Imaging, University of Groningen, University Medical Centre Groningen, Groningen, The Netherlands; 3 Department of Clinical Pharmacy and Pharmacology, University of Groningen, University Medical Centre Groningen, Groningen, The Netherlands; 4 Department of Pathology and Medical Biology, University of Groningen, University Medical Centre Groningen, Groningen, The Netherlands; 5 Department of Medical Oncology, Erasmus MC Cancer Institute, Rotterdam, The Netherlands

**Keywords:** ^89^Zr-trastuzumab PET, Breast cancer, Human epidermal growth factor receptor 2, Clinical decision making

## Abstract

**Background:**

Up-to-date information on human epidermal growth factor receptor 2 (HER2) status in breast cancer (BC) is important, as expression can vary during the course of the disease, necessitating anti-HER2 therapy adjustments. Repeat biopsies, however, are not always possible. In this feasibility trial we assessed whether ^89^Zr-trastuzumab PET could support diagnostic understanding and aid clinical decision making, when HER2 status could not be determined by standard work up. Additionally, HER2 status on circulating tumour cells (CTCs) was assessed.

**Patients and methods:**

^89^Zr-trastuzumab PET was performed in patients if disease HER2 status remained unclear after standard work up (bone scan, ^18^F-FDG PET, CT and if feasible a biopsy). PET result and central pathologic revision of available tumour biopsies were reported to the referring physician. CTC HER2 status prior to PET was evaluated afterwards and therefore not reported. Diagnostic understanding and treatment decision questionnaires were completed by the referring physicians before, directly after and ≥ 3 months after ^89^Zr-trastuzumab PET.

**Results:**

Twenty patients were enrolled: 8 with two primary cancers (HER2-positive and HER2-negative BC or BC and non-BC), 7 with metastases inaccessible for biopsy, 4 with prior HER2-positive and -negative metastases and 1 with primary BC with equivocal HER2 status. ^89^Zr-trastuzumab PET was positive in 12 patients, negative in 7 and equivocal in 1 patient. In 15/20 patients, ^89^Zr-trastuzumab PET supported treatment decision. The scan altered treatment of 8 patients, increased physicians’ confidence without affecting treatment in 10, and improved physicians’ disease understanding in 18 patients. In 10/20 patients CTCs were detected; 6/10 showed HER2 expression. CTC HER2 status was not correlated to ^89^Zr-trastuzumab PET result or treatment decision.

**Conclusion:**

^89^Zr-trastuzumab PET supports clinical decision making when HER2 status cannot be determined by standard work up. The impact of CTC HER2 status needs to be further explored.

**Electronic supplementary material:**

The online version of this article (10.1007/s00259-018-4099-8) contains supplementary material, which is available to authorized users.

## Introduction

In metastatic breast cancer, treatment options are largely dependent upon the presence of the oestrogen receptor, progesterone receptor and human epidermal growth factor receptor 2 (HER2), in addition to tumour load and location. The outcome of HER2 positive metastatic disease has fundamentally improved since the development of effective HER2 targeting agents such as trastuzumab, pertuzumab and trastuzumab-emtansine [1]. In this light, it is of particular interest that HER2 status can change during disease course, consequently necessitating anti-HER2 therapy adjustment. Furthermore, HER2 status discordancy between primary and residual or metastatic lesions, either HER2 loss or gain [2], was related to shorter disease-free and overall patient survival in retrospective [3, 4] and prospective analyses [5]. This discordancy, measured by immunohistochemistry (IHC) and/or in situ hybridization (ISH) techniques, ranged between 0 and 33% [2, 3, 6–14]. Moreover, HER2 expression can be heterogeneous within the same tumour [6, 15, 16]. Therefore, temporal and spatial heterogeneity may fundamentally affect HER2 status and therefore treatment response. Based on this data, clinical guidelines encourage repeat biopsies during the course of the disease. However, due to technical or patient related factors, tumour lesions are not always (safely) accessible, leaving the clinician with a dilemma with regard to the disease’s HER2 status.

HER2 imaging using ^89^Zr-trastuzumab positron emission tomography (PET) could be a strategy to noninvasively assess HER2 expression in tumour lesions throughout the whole body [17, 18]. It might, therefore, become a valuable tool to guide clinical decision making in metastatic breast cancer patients, who—despite extensive work-up—pose a clinical dilemma [19, 20]. Characterization of circulating tumour cells (CTCs) might be another patient-friendly method to assess HER2 status on metastatic cells [21]. Since CTCs are likely shed from different tumour sites—metastases and the primary tumour, if still present—they might reflect both HER2 status and tumour heterogeneity. Consequently, the aim of this clinical feasibility trial was to assess whether ^89^Zr-trastuzumab PET supports clinical decision making in patients suspected of metastatic or locally recurrent HER2-positive breast cancer, presenting with a dilemma defined as failure of the standard work-up to evaluate the present HER2 status of their disease. In addition, HER2 status of CTCs was assessed and correlated to treatment decision and ^89^Zr-trastuzumab PET result.

## Patients and methods

### Patient population

This prospective single-centre clinical trial protocol was approved by the medical ethics committee of the University Medical Centre Groningen (UMCG; ClinicalTrials.gov identifier NCT01832051). All patients provided written informed consent.

Patients with suspected metastatic disease or local recurrence of HER2-positive breast cancer with a clinical dilemma defined as failure of standard work-up to evaluate the HER2 status were eligible. HER2-positivity, reported in the patient’s history, was defined positive with an IHC of score 3+ or IHC of score 2+ followed by ISH showing HER2 amplification according to the American Society of Clinical Oncology guidelines [22]. Standard imaging work-up preferably consisted of a computed tomography (CT) of the chest and abdomen, a bone scintigraphy and a fluorine-18-fluorodeoxyglucose (^18^F-FDG) PET scan, accompanied by a metastasis biopsy, if feasible. Other eligibility criteria included age ≥ 18 years and Eastern Cooperative Oncology Group performance status of 0–2. Patients with a history of allergic reactions to immunoglobulins and pregnant or lactating women, as well as patients with any inabilities not allowing compliance with the study procedures, were excluded.

### ^89^Zr-trastuzumab PET scan

Clinical grade ^89^Zr-trastuzumab was produced at the UMCG as described previously [17]. Patients received 37 MBq (± 10%; ~1 m Ci) ^89^Zr-trastuzumab intravenously supplemented with unlabeled antibody to a total amount of 50 mg trastuzumab. Four days postinjection, head to upper thigh was scanned in up to nine bed positions with 5 min/bed position in combination with a low dose CT scan for attenuation correction and anatomic reference with a Biograph mCT 64-slice PET/CT camera (Siemens). PET scans were reconstructed and visually analysed by one dedicated nuclear medicine physician. The ^89^Zr-trastuzumab PET scan was considered positive, when in comparison to the ^18^F-FDG PET and in conjunction with conventional imaging (e.g. contrast enhanced CT scan, bone scan or MRI in case of brain metastases) the entire tumour load or a dominant part of the tumour load showed ^89^Zr-trastuzumab tumour uptake [23]. ^89^Zr-trastuzumab tumour uptake was considered substantial when tumour tracer uptake in visceral lesions (excluding brain) was at least comparable to or higher than liver background or in case of brain metastases when ^89^Zr-trastuzumab uptake was exceeding brain background uptake allowing clear identification of the metastasis. Interpretation of ^89^Zr-trastuzumab uptake in bone lesions was assessed in relation with visceral metastases.

Retrospectively, PET images were reconstructed using the harmonized reconstruction algorithm recommended for multicentre ^89^Zr-mAb PET scan trials [24] and all tumour lesions on the conventional imaging were recorded, including measurability according to RECIST 1.1 [25] and prior radiation therapy. Tumour lesions with a diameter of >15 mm on contrast enhanced CT scan were quantified, when tumour tracer uptake was considered not to be influenced by surrounding tissue and when a lesion was not irradiated ≤6 months of the ^89^Zr-trastuzumab PET scan. With the AMIDE (A Medical Image Data Examiner) software (version 0.9.3, [26]) radioactivity was quantified in manually drawn volumes of interest around tumour lesions and several background organs, and standardized uptake values (SUV) were calculated. We report SUVmax for tumour lesions and SUVmean for normal organ tracer uptake.

### Clinical value

To assess the influence of the ^89^Zr-trastuzumab PET scan on treatment decision, referring physicians completed earlier validated questionnaires before, directly after and > 3 months after the ^89^Zr-trastuzumab PET scan [27]. Information on the patient’s history, which dilemma incited the referral for ^89^Zr-trastuzumab PET, as well as the intended treatment were assessed with the first questionnaire. In the second questionnaire, completed after receiving the scan result, the treating physician was asked to give the final diagnosis, the chosen treatment strategy and information on potential additional tests planned. With the last questionnaire, referring physicians were asked to rate the contribution of the ^89^Zr-trastuzumab PET scan to their diagnostic understanding of the patient’s disease and the choice of therapy using a 5-point scale (Supplementary Table S1). All questionnaires were checked for internal consistency.

### Archival tumour samples

Available archival tumour samples from the primary tumour site(s) or metastases were centrally revised and IHC (SP3; rabbit monoclonal antibody; NeoMarkers, Lab Vision Corp., Thermo Fisher Scientific, Fremont, California, USA), and in case of an IHC 2+ score ISH (PathVysion HER2/neu DNA probe kit, Vysis, Abbott Molecular, Des Plaines, IL) were repeated. HER2 positivity was defined as IHC 3+, or IHC 2+ with a positive ISH (HER2:CEP17 ratio ≥ 2.0 or an average of ≥6.0 HER2 copies per nucleus; [22]).

### Circulating tumour cell analysis

Before tracer injection, blood for CTC enumeration and CTC HER2 expression analysis was collected. Samples were transported to the laboratory of Clinical Tumour Immunology, Erasmus MC Cancer Institute, Rotterdam, the Netherlands, for analysis. One CellSave tube was used to obtain an EpCAM-based CTC count from 7.5 mL blood using the Epithelial Cell Kit (Janssen Diagnostics LLC, Raritan, NJ, USA) on CellSearch System according to the manufacturer’s instructions. CTCs were further characterized for HER2 expression within the Cell-Search system by a FITC-labelled anti-HER2 antibody as described by the manufacturer (CellSearch tumour phenotyping reagent HER2/neu; Janssen Diagnostics LLC). HER2 immunofluorescence staining intensity of 3+ and 2+ were scored as HER2-positive as described earlier [28]. CTC HER2 status was evaluated after inclusion of all patients and was not reported to the referring physician.

### Statistical analysis

Statistical analyses were performed using SPSS Version 23. To assess relation between CTC result and ^89^Zr-trastuzumab scan result or chosen treatment strategy, Spearman’s correlation was used. *P* ≤ 0.05 was considered to be a significant difference. Data are presented as mean ± standard deviation (SD), unless otherwise stated.

## Results

### Patient characteristics

Twenty patients were enrolled between July 2013 and June 2015 from all over the Netherlands and the Northern border area of Germany, with a median distance to our centre of 125 km (range 20–247, Table 1). The ^89^Zr-trastuzumab PET scan was requested by the referring physicians (all: medical oncologists) due to following reasons (Supplementary Table S2): (i) To differentiate between metastases of two primary cancers, either two primary breast cancers (one HER2-positive and the other HER2-negative), or a HER2-positive breast cancer and a second primary cancer from another origin (*N* = 8), (ii) to assess HER2 status of a single lesion inaccessible for biopsy, or in case of multiple lesions inability to perform repeat biopsies (*N* = 7), (iii) to assess HER2 expression of metastatic breast cancer with known heterogeneous HER2 status over time (*N* = 4), and (iv) to evaluate HER2 expression in metastatic breast cancer with prior equivocal histopathological result (HER2 IHC score 2+, ISH result: average 4.23 HER2 gene copies/nucleus, *N* = 1).

**Table 1:** Patient characteristics

Characteristic	All patients (N = 20)
Median age, y (range)	56 (37–71)
Median travel distance to facility with ^89^Zr-trastuzumab PET, km (range)	125 (20–247)
Sex	
Male	0
Female	20
Prior lines of anti-HER2 therapy	
0	4
1	9
2	2
> 3	5
Reported main clinical dilemma	
Two primary cancers	8
Unfeasibility of (repeated) biopsy	7
Heterogeneous HER2 status over time	4
Equivocal histopathological workup	1

### ^89^Zr-trastuzumab PET

The highest normal organ ^89^Zr-trastuzumab uptake was observed in the liver, followed by the kidney, intestine (=faeces), blood pool and the spleen; the lowest was seen in subcutaneous tissue and the brain (Supplementary Figure S1).

**Fig. 1:**
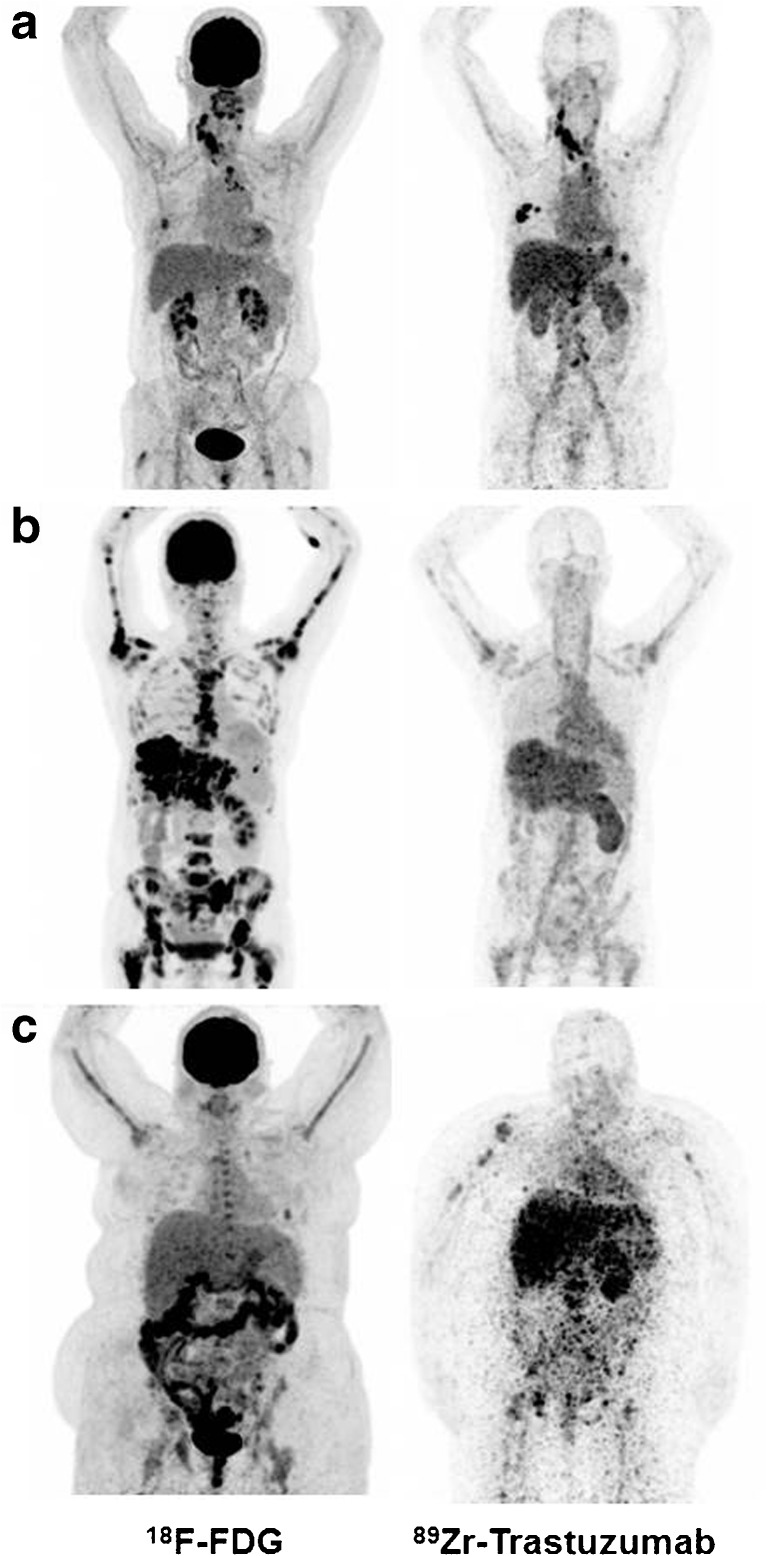
^18^F-FDG (*left*) and ^89^Zr-trastuzumab PET scans (*right*) of three patients: Example of a patient with a ^89^Zr-trastuzumab PET scan considered HER2-positive (**a**), a ^89^Zr-trastuzumab PET scan considered HER2-negative (**b**) and an ^89^Zr-trastuzumab PET scan considered equivocal (**c**)

At visual assessment, ^89^Zr-trastuzumab tumour uptake was considered positive in 12 patients, negative in seven patients and equivocal in one patient (Fig. 1 and Supplementary Table S2).

Retrospectively, a total of 404 tumour lesions were delineated on ^89^Zr-trastuzumab PET after primary visual assessment of which 264 (65%) were considered evaluable. In two patients, none of the known metastases appeared on ^89^Zr-trastuzumab PET and their scans, therefore, were considered negative. In the remaining 18 patients a median of 9 lesions (range 1–69) was evaluable. Heterogeneity of tumour tracer uptake was observed within patients, with a maximal 8-fold difference within one patient. Also, tumour tracer uptake varied greatly between patients, with a maximal 13-fold difference (data not shown).

### HER2 status in tumour biopsies in comparison to ^89^Zr-trastuzumab PET

For central revision a total of 42 tumour samples of 20 patients were available (primary *N* = 18, secondary *N* = 10, metastasis *N* = 14). One patient, who had reported HER2-positive disease, was diagnosed with heterogeneous disease after central pathology revision (Table 2). Furthermore, two out of ten patients with a reported combination of HER2-positive and HER2-negative disease and the one patient with the equivocal histopathological result were diagnosed with HER2-negative disease after central revision.

**Table 2:** Reported HER2 status in biopsies of primary tumours and metastases versus result of central pathology revision, and ^89^Zr-trastuzumab PET result

Patient	Reported HER2 status	HER2 status after central revision	^89^Zr-trastuzumab PET result
1	HER2+ and HER2-	HER2+ and HER2-	Positive
2	HER2+	HER2+	Positive
3	HER2+ and HER2-	HER2-^a^	Negative
4	HER2+ and HER2-	HER2+ and HER2-	Positive
5	HER2+	HER2+	Negative^b^
6	HER2+	HER2+	Positive
7	HER2+ and HER2-	HER2+ and HER2-	Equivocal
8	HER2+	HER2+	Positive
9	HER2+ and HER2-	HER2+ and HER2-	Negative
10	HER2+	HER2+	Positive
11	HER2+ and HER2-	HER2+ and HER2-	Positive
12	HER2+ and HER2-	HER2+ and HER2-	Negative
13	HER2+ and HER2-	HER2+ and HER2-	Positive
14	HER2+ and HER2-	HER2-^a^	Negative
15	HER2+	HER2+	Positive
16	HER2+	HER2+	Positive
17	HER2+ and HER2-	HER2+ and HER2-	Negative
18	equivocal	HER2-	Negative
19	HER2+	HER2+ and HER2-^a^	Positive
20	HER2+	HER2+	Positive

^a^Initial HER2 IHC interpretation of primary tumour biopsy false positive

^b^Leptomeningeal metastases visualized on MRI where not visible on ^89^Zr-trastuzumab PET either due to negative HER2 status or due to their size below the detection limit

The ^89^Zr-trastuzumab PET scan was positive in seven out of eight patients with a previously HER2 positive primary tumour, and in five out of nine patients with a previous combination of HER2-positive and HER2-negative disease according to available tumour tissue (Table 2).

### Clinical value of ^89^Zr-trastuzumab PET

**Fig. 2:**
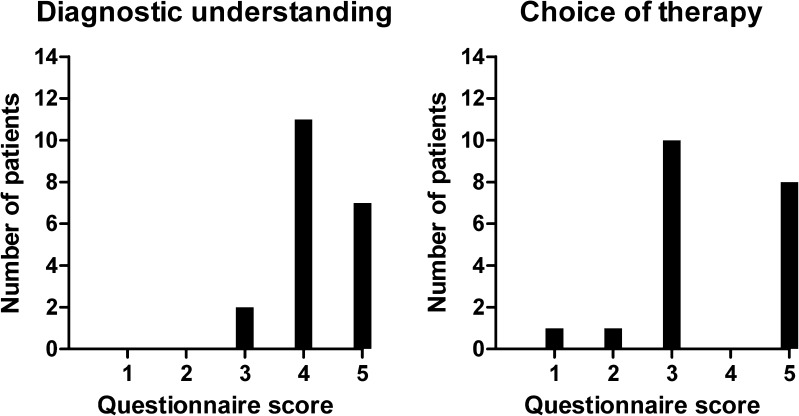
Contribution of the ^89^Zr-trastuzumab PET scan to the treating physicians diagnostic understanding (*left*) and choice of therapy (*right*) using a 5-point scale

The work-up including ^89^Zr-trastuzumab PET scan improved the treating physician’s understanding of the patient’s disease in 18 (90%) patients (Fig. 2). The confidence over the (unaltered) treatment choice was improved in ten patients (50%), and in eight patients (40%) the treatment was changed. Five patients were started on anti-HER2 treatment and three patients did not receive HER2-targeting agents as a consequence of the ^89^Zr-trastuzumab scan (Table 3). In one patient the scan did not influence the understanding and/or treatment choice, and one physician of a patient with osteosarcoma and simultaneous HER2-positive breast cancer, rated choice of treatment based on the ^89^Zr-trastuzumab PET as non-beneficial for the patient, although the scan improved her understanding of the disease.

**Table 3:** Treatment decision before and after ^89^Zr-trastuzumab PET

Treatment planned before ^89^Zr-trastuzumab PET	Treatment given after ^89^Zr-trastuzumab PET		
	Anti-HER2 (± chemo)	No anti-HER2 (other systemic treatment)	No systemic treatment
• Anti-HER2 (± chemo)	5	2	0
• No anti-HER2 (other systemic treatment)	2	4	0
• No systemic treatment	1	0	3
• Unsure on systemic treatment choice/dependent on additional test	2	1	0

### CTC HER2 status

CTCs were found in half of the patient population (median number of CTCs/7.5 mL = 6.5, range 1–99). In six of them, HER2-positive CTCs were found and three of the six patients also had a positive ^89^Zr-trastuzumab PET scan (Supplementary Table S3). Two out of the six patients, both with positive ^89^Zr-trastuzumab PET, received anti-HER2 treatment subsequently. Overall, CTC result was not correlated to ^89^Zr-trastuzumab PET result or subsequent treatment decision (correlation with PET result: *r* = 0.074, *P* = 0.84; correlation with treatment decision: *r* = −0.37, *P* = 0.92).

## Discussion

In this small prospective clinical feasibility trial we show for the first time that ^89^Zr-trastuzumab PET can support diagnostic understanding and clinical decision making when HER2 status of metastatic or locally recurrent breast cancer cannot be determined by standard work up.

The ^89^Zr-trastuzumab PET scan improved the physician’s understanding of the patient’s disease in the majority of patients and the treatment strategy was changed in 40% of the study population. Five patients received initially unplanned anti-HER2 therapy, whereas in three patients, intended anti-HER2 therapy was withheld. By doing this, the latter patients were possibly saved from toxicity of a potentially ineffective treatment. Moreover, the savings of treatment-related costs outweigh scan-related costs manifold. Thereby, distance to ^89^Zr-trastuzumab PET was no issue in our trial as patients were willing to travel up to almost 250 km (~150 miles), implying that such molecular scan techniques, although localized only in specialized centres, can be within reach of a vast majority of patients. Using additional molecular imaging in standard clinical care will increase radiation exposure. In case of a ^89^Zr-trastuzumab PET, this additional radiation exposure equals that of one diagnostic CT scan of the chest, abdomen and pelvis [29–32]. The balance between risks and benefits of any additional procedure should always be carefully considered in any patient population. In this particular population, a diagnostic dilemma is known to negatively affect their survival if left unsolved. In light of the potentially helpful information gained by the scan and also considering the incurable nature of their disease, we think that the benefits of a ^89^Zr-trastuzumab PET outweigh the risks in this particular patient population. Therefore, we consider this scan as suitable for clinical practice.

CTC analysis in metastatic breast cancer has shown to be a strong prognostic factor [33–36]. Since CTCs probably originate from different tumour sites, they might also provide a comprehensive view of tumour characteristics like HER2 status, including tumour heterogeneity. In our trial, CTCs were only detected in half of the patients, which corresponds with the earlier reported CTC detection rate [36]. The impact of CTC HER2 status on clinical decision making is unclear from the present study, as the result was not reported to the referring physician. Therefore this will have to be further explored. However, central pathology revision including renewed HER2 staining, and subsequent comparison of primary tumour and metastases biopsies, did deliver new insights in HER2 status in three out of 20 patients in this study. Therefore this could be worth considering in the standard setting.

Validation of molecular scan techniques is still an ongoing process. Clinical utility of ^89^Zr-trastuzumab PET, especially the relation of scan results with treatment response and survival data in recently diagnosed metastatic breast cancer patients, is currently assessed in a prospective, multicentre observational cohort study conducted in the Netherlands (ClinicalTrials.gov Identifier: NCT01957332). In this trial, intra-patient heterogeneity of tumour tracer uptake will also further be evaluated, as so far the clinical implication of the observed heterogeneity is unclear. The trial, furthermore, supports validation and standardization of interpretation of this PET imaging technique, which is instrumental for potential further wider application as possible biomarker for treatment response in the future. Additionally, the impact of CTC enumeration and characterization for HER2 and its relation with ^89^Zr-trastuzumab PET is further explored in the mentioned trial. However, the present study already establishes ^89^Zr-trastuzumab PET as a diagnostic tool to help the treating physician in clinical decision making, in this niche population of patients with an otherwise undetermined HER2 status of their disease.

## Electronic supplementary material

Supplementary table S1 (DOCX 15 kb)

Supplementary table S2 (DOCX 23 kb)

Supplementary table S3 (DOCX 20 kb)

Supplementary figure S1. Normal organ ^89^Zr-trastuzumab distribution depicted as mean SUVmean (+SD) (JPG 542 kb)
