# Supplementary material for: 89Zr-trastuzumab PET supports clinical decision making in breast cancer patients, when HER2 status cannot be determined by standard work up
Source: Eur J Nucl Med Mol Imaging. 2018 Jul 30;45(13):2300–6. doi: 10.1007/s00259-018-4099-8 (PMC6208812; doi:10.1007/s00259-018-4099-8)
Supplement: Supplementary file 1 — (DOCX 15 kb) [file 259_2018_4099_MOESM1_ESM.docx]

**Table S1.** Questionnaire assessing clinical value of ^89^Zr-trastuzumab PET

| Diagnostic understanding | |
| --- | --- |
| 1 | ^89^Zr-trastuzumab PET confused my understanding of this patient’s disease and led to investigations I would not otherwise have done. |
| 2 | ^89^Zr-trastuzumab PET confused my understanding of this patient’s disease but did not lead to any additional investigations. |
| 3 | ^89^Zr-trastuzumab PET had little or no effect on my understanding of this patient’s disease. |
| 4 | ^89^Zr-trastuzumab PET provided information which substantially improved my understanding of this patient’s disease. |
| 5 | My understanding of this patient’s disease depended upon diagnostic information provided only by ^89^Zr-trastuzumab PET (unavailable from any other non-surgical procedure). |
| Choice of therapy | |
| 1 | ^89^Zr-trastuzumab PET led me to choose therapy which in retrospect was not in the best interests of the patient. |
| 2 | ^89^Zr-trastuzumab PET was of no influence in my choice of therapy. |
| 3 | ^89^Zr-trastuzumab PET did not alter my choice of therapy but did increase my confidence in the choice. |
| 4 | ^89^Zr-trastuzumab PET contributed to a change in my chosen therapy but other factors (other imaging tests, other diagnostic tests, changes in patient status) were equally or more important. |
| 5 | ^89^Zr-trastuzumab PET was very important compared with other factors in leading to a beneficial change in therapy. |
