# Supplementary material for: 89Zr-trastuzumab PET supports clinical decision making in breast cancer patients, when HER2 status cannot be determined by standard work up
Source: Eur J Nucl Med Mol Imaging. 2018 Jul 30;45(13):2300–6. doi: 10.1007/s00259-018-4099-8 (PMC6208812; doi:10.1007/s00259-018-4099-8)
Supplement: Supplementary file 2 — (DOCX 23 kb) [file 259_2018_4099_MOESM2_ESM.docx]

**Table S2.** Details on the clinical dilemma and the results per scan modality per patient

| Patient | Dilemma  category | Description of dilemma | Bone scan | CT scan | ^18^F-FDG PET-positive lesions | ^89^Zr-trastuzumab PET interpretation |
| --- | --- | --- | --- | --- | --- | --- |
| 1 | Heterogeneous HER2 status over time | HER2+ primary breast cancer and HER2- metastasis during course of disease | Two bone metastases (femur) | Peritonitis carcinomatosa, soft tissue metastases chest and back | Peritonitis carcinomatosa, multiple malignant lymph nodes (axillary, mediastinal), soft tissue metastases chest and back including subcutaneous lesions, four bone metastases (femur and rib), suspicious uptake intrapulmonal | Dominant part of tumour load shows ^89^Zr-trastuzumab uptake exceeding healthy liver background  🡺 scan considered positive |
| 2 | Evaluation of HER2 status | Doubtful whether continuation of anti-HER2 therapy is indicated in patient with treatment induced cardiac toxicity | No bone metastases | Multiple lymph nodes (cervical, mediastinal), inhomogeneous thyroid gland, multiple skin metastases | Multiple lymph nodes (cervical, mediastinal, abdominal), multiple skin metastases, mammary lesion, right thyroid gland | Entire tumour load shows ^89^Zr-trastuzumab uptake exceeding healthy liver background  🡺 scan considered positive |
| 3 | Heterogeneous HER2 status over time | HER2+ primary breast cancer and HER2- metastasis during course of disease | Multiple bone metastases (skull, sternum, clavicle, pelvic bone, femur, spine, ribs) | Multiple liver metastases, multiple bone metastases | Multiple bone metastases (skull, sternum, clavicle, pelvic bone, femur, spine, ribs), multiple liver metastases, multiple lymph nodes (cervical, mediastinal, iliacal) | Only slight ^89^Zr-trastuzumab uptake in all for metastases suspicious lesions, uptake less than healthy liver background  🡺 scan considered negative |
| 4 | Heterogeneous HER2 status during time | HER2- primary breast cancer and HER2+ metastasis during course of disease | No bone metastases | Multiple lymph nodes (cervical, mediastinal, axillary), multiple lung metastases, two liver metastases | Multiple lymph nodes (cervical, mediastinal, axillary, abdominal), multiple lung metastases, two liver metastases, single bone metastasis | ^89^Zr-trastuzumab uptake in cervical lymph node and liver metastases exceeds healthy liver background, remainder lesions only slight tracer uptake  🡺 scan considered positive |
| 5 | Evaluation of HER2 status | Leptomeningeal metastases visualized on MRI*, biopsy not possible | No bone metastases | No suspicious lesions | No suspicious lesions | No focal ^89^Zr-trastuzumab uptake  🡺 scan considered negative |
| 6 | Two primary tumours | Differentiation between lesions of HER2+ breast cancer and malignant lymphoma | Single bone metastasis (scapula) | Multiple liver metastases | Diffuse liver metastases, diffuse ^18^F-FDG uptake in bone marrow | Entire tumour load shows ^89^Zr-trastuzumab uptake exceeding healthy liver background  🡺 scan considered positive |
| 7 | Two primary tumours | HER2+ primary breast cancer and HER2- secondary breast cancer | Multiple bone metastases (diffuse in whole skeleton) | Multiple bone metastases, suspicious lesion adrenal gland and single lung nodule | No clear evidence of bone metastases, lesion in adrenal gland or lung, only diffuse ^18^F-FDG uptake | Diffuse ^89^Zr-trastuzumab uptake in suspicious bone and visceral lesions, uptake less than healthy liver background  🡺 scan considered equivocal |
| 8 | Evaluation of HER2 status | Doubtful whether continuation of anti-HER2 therapy is indicated after years of treatment and progression of disease | Multiple bone metastases (spine, ribs, humerus, scapula, pelvic bone, femur) | Multiple bone metastases (spine, ribs, humerus, scapula, pelvic bone, femur) | Multiple bone metastases (spine, ribs, humerus, scapula, pelvic bone, femur), multiple brain metastases | Dominant part of tumour load shows ^89^Zr-trastuzumab uptake exceeding healthy liver background  🡺 scan considered positive |
| 9 | Two primary tumours | HER2+ primary breast cancer, HER2- breast cancer metastases and thyroid cancer | Multiple bone metastases (ribs) | Multiple bone metastases (ribs) | Multiple bone metastases (ribs) | Only slight ^89^Zr-trastuzumab uptake in all for metastases suspicious lesions, uptake less than healthy liver background  🡺 scan considered negative |
| 10 | Evaluation of HER2 status | Discrepant response to anti-HER2 therapy, doubtful whether continuation of anti-HER2 therapy is indicated | Single bone metastasis (spine) | Single bone metastasis (spine) | NA | Bone metastasis shows ^89^Zr-trastuzumab uptake exceeding healthy liver background  🡺 scan considered positive |
| 11 | Two primary tumours | HER2- primary breast cancer and HER2+ secondary breast cancer | NA | Multiple lymph nodes (cervical, mediastinal) | Multiple lymph nodes (cervical, mediastinal) | Dominant part of tumour load shows ^89^Zr-trastuzumab uptake exceeding healthy liver background  🡺 scan considered positive |
| 12 | Two primary tumours | HER2- primary breast cancer and HER2+ secondary breast cancer | Multiple bone metastases (pelvic bone, spine, scapula, femur) | Multiple bone metastases (pelvic bone, spine, scapula, femur), multiple liver metastases | Multiple bone metastases (pelvic bone, spine, scapula, femur), multiple liver metastases, multiple suspicious lymph nodes (axillary) | No focal ^89^Zr-trastuzumab uptake  🡺 scan considered negative |
| 13 | Two primary tumours | HER2+ primary breast cancer and HER2- secondary breast cancer | No bone metastases | Lymph node (mediastinal) and single bone metastasis (sacrum) | Lymph node (mediastinal) and single bone metastasis (sacrum) | Entire tumour load shows ^89^Zr-trastuzumab uptake exceeding healthy liver background  🡺 scan considered positive |
| 14 | Heterogeneous HER2 status over time | HER2+ primary breast cancer and HER2- metastasis during course of disease | Multiple bone metastases (sternum, pelvic bone) | Multiple bone metastases (sternum, pelvic bone), multiple lymph nodes (cervical, mediastinal), pleural metastases, multiple liver metastases | Multiple bone metastases (sternum, pelvic bone), multiple lymph nodes (cervical, mediastinal), pleural metastases, multiple liver metastases | Only slight ^89^Zr-trastuzumab uptake in all for metastases suspicious lesions, uptake less than healthy liver background  🡺 scan considered negative |
| 15 | Evaluation of HER2 status | Suspicious lymph node, no biopsy possible | No bone metastases | Multiple lymph nodes (abdominal), multiple liver metastases | Multiple lymph nodes (abdominal), multiple liver metastases | Dominant part of tumour load shows ^89^Zr-trastuzumab uptake exceeding healthy liver background  🡺 scan considered positive |
| 16 | Evaluation of HER2 status | Differentiation between radionecrosis and recurrent brain metastasis | No bone metastases | No suspicious lesions | Single brain metastasis | Brain metastasis shows ^89^Zr-trastuzumab uptake exceeding healthy liver and brain background  🡺 scan considered positive |
| 17 | Two primary tumours | HER2- primary breast cancer and HER2+ secondary breast cancer | Diffuse uptake in multiple locations in the spine (differential diagnosis osteoporosis or metastases) | Suspicious bone lesions (spine, differential diagnosis osteoporosis or metastases) | Multiple lymph nodes (cervical, mediastinal), diffuse uptake in spinal lesions (differential diagnosis osteoporosis or metastases) | Only slight ^89^Zr-trastuzumab uptake in all for metastases suspicious lesions, uptake less than healthy liver background  🡺 scan considered negative |
| 18 | Equivocal or ambiguous work-up | HER2 ISH equivocal | Multiple bone lesions (sternum, scapula, ribs, spine, pelvic bone, femur) | Mamma, multiple lymph nodes (axillary, mediastinal, abdominal), multiple bone lesions (sternum, scapula, ribs, spine, pelvic bone, femur) | Mamma, multiple lymph nodes (axillary, mediastinal), multiple bone lesions (sternum, scapula, ribs, spine, pelvic bone, femur) | Only slight ^89^Zr-trastuzumab uptake in all for metastases suspicious lesions, uptake less than healthy liver background  🡺 scan considered negative |
| 19 | Two primary tumours | HER2+ breast cancer and osteosarcoma | Single bone metastases (rib) | Subcutaneous thoracic lesion and single bone metastases (rib), single pleural lesion, multiple lymph nodes (parasternal) | Subcutaneous thoracic lesion and single bone metastases (rib), multiple lymph nodes (parasternal), single pleural lesion (suspicious for osteosarcoma) | Dominant part of tumour load shows ^89^Zr-trastuzumab uptake exceeding healthy liver background, no to slight ^89^Zr-trastuzumab uptake in pleural lesion  🡺 scan considered positive |
| 20 | Evaluation of HER2 status | Suspicious lung nodule, no biopsy possible | No bone metastases | Suspicious pulmonary lesions (differential diagnosis infiltrates, metastases), multiple bone lesions (spine, pelvic bone, ribs) | Two aspecific lesions mamma, multiple suspicious intrapulmonary lesions, single brain metastasis, single bone metastasis (pelvic bone) | Brain metastasis shows ^89^Zr-trastuzumab uptake exceeding healthy liver and brain background, remaining lesions don’t show evident ^89^Zr-trastuzumab uptake  🡺 scan considered positive |

* Three extra axial lesions visualized on MRI brain, differential diagnosis leptomeningeal metastases or meningioma. NA, not available.
