# Supplementary material for: 89Zr-trastuzumab PET supports clinical decision making in breast cancer patients, when HER2 status cannot be determined by standard work up
Source: Eur J Nucl Med Mol Imaging. 2018 Jul 30;45(13):2300–6. doi: 10.1007/s00259-018-4099-8 (PMC6208812; doi:10.1007/s00259-018-4099-8)
Supplement: Supplementary file 3 — (DOCX 20 kb) [file 259_2018_4099_MOESM3_ESM.docx]

**Table S3.** Results of ^89^Zr-trastuzumab PET *versus* CTC count and CTC HER2 status

| Patient | ^89^Zr-trastuzumab PET result | CTC count (*N*) | HER2-positive CTCs (*N*) |
| --- | --- | --- | --- |
| 1 | Positive | 8 | 0 |
| 2 | Positive | 5 | 3 |
| 3 | Negative | 70 | 0 |
| 4 | Positive | 0 | - |
| 5 | Negative | 0 | - |
| 6 | Positive | 99 | 94 |
| 7 | Equivocal | 0 | - |
| 8 | Positive | 5 | 5 |
| 9 | Negative | 21 | 1 |
| 10 | Positive | 0 | - |
| 11 | Positive | 0 | - |
| 12 | Negative | 13 | 2 |
| 13 | Positive | 0 | - |
| 14 | Negative | 1 | 1 |
| 15 | Positive | 1 | 0 |
| 16 | Positive | 0 | - |
| 17 | Negative | 0 | - |
| 18 | Negative | 0 | - |
| 19 | Positive | 1 | 0 |
| 20 | Positive | 0 | - |
