# Supplementary figures and images for: 89Zr-trastuzumab PET supports clinical decision making in breast cancer patients, when HER2 status cannot be determined by standard work up
Source: Eur J Nucl Med Mol Imaging. 2018 Jul 30;45(13):2300–6. doi: 10.1007/s00259-018-4099-8 (PMC6208812; doi:10.1007/s00259-018-4099-8)

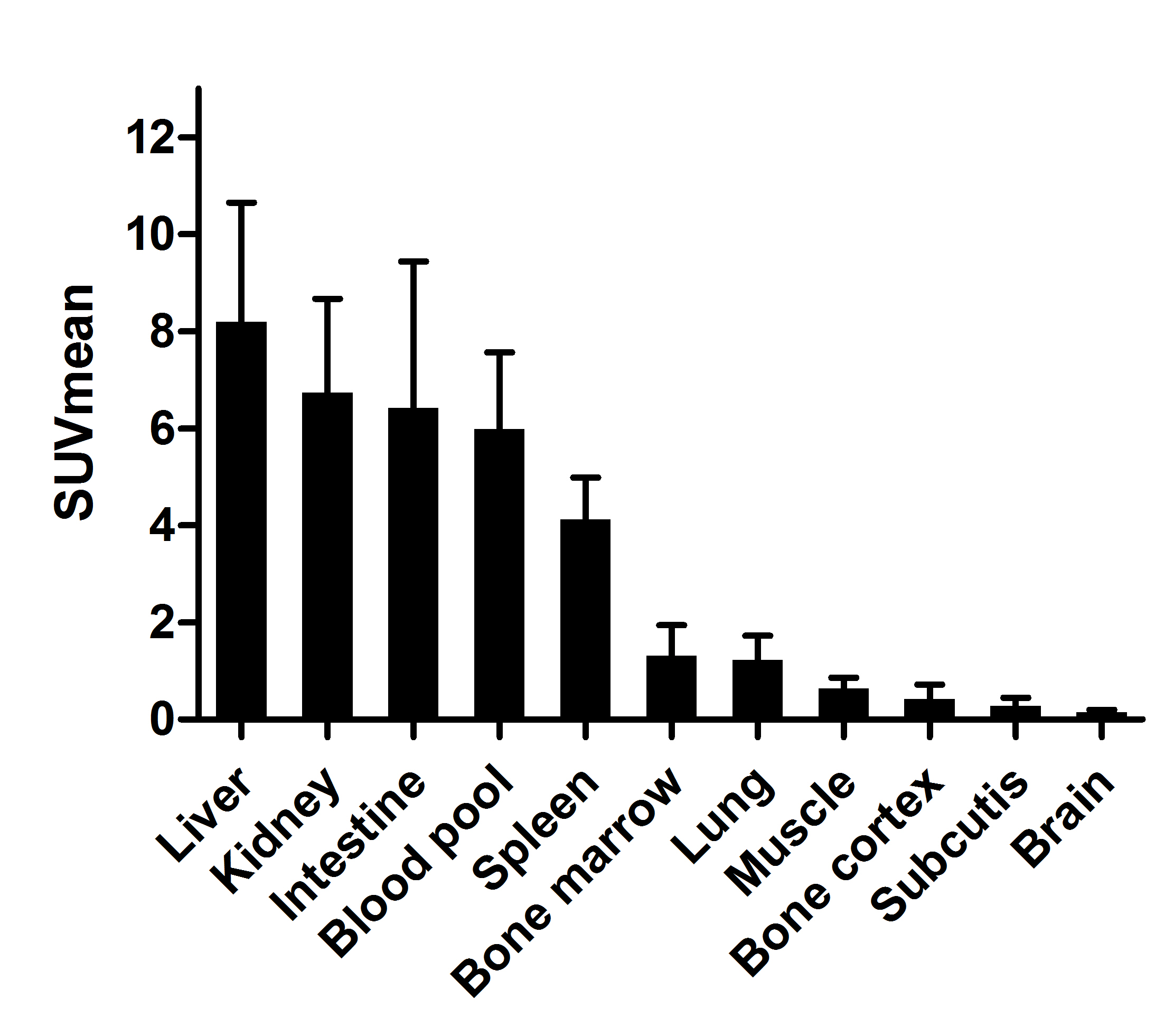

Supplement: Supplementary file 4 — Normal organ 89Zr-trastuzumab distribution depicted as mean SUVmean (+SD) (JPG 542 kb) [file 259_2018_4099_MOESM4_ESM.jpg]
